# Clinical and Hormonal Determinants of Propofol Requirement During Oocyte Pick-Up: A Prospective Observational Study

**DOI:** 10.3390/jcm15114280

**Published:** 2026-06-01

**Authors:** Gözde Gürsoy Çirkinoğlu, Tuba Kuvvet Yoldaş, Aylin Ateşalp, Halide Hande Şahinkaya, Zeki Tuncel Tekgül

**Affiliations:** 1 Department of Anesthesiology and Reanimation, Izmir City Hospital, 35540 Izmir, Türkiye; 2 Department of Anesthesiology and Reanimation, University of Health Sciences Izmir Faculty of Medicine, 35100 Izmir, Türkiye

**Keywords:** propofol, estradiol, progesterone, oocyte pick-up, in vitro fertilization, anesthetic requirement

## Abstract

**Objectives:** Oocyte pick-up (OPU) is commonly performed under propofol-based sedation during in vitro fertilization (IVF). However, considerable interindividual variability in propofol requirement has been observed. Controlled ovarian hyperstimulation results in supraphysiological levels of ovarian steroid hormones, which may influence anesthetic sensitivity. This study aimed to evaluate the relationship between preprocedural serum estradiol and progesterone levels and propofol requirement during OPU performed under bispectral index (BIS)-guided sedation. **Methods:** In this prospective observational study, 96 women undergoing OPU were included. Serum estradiol and progesterone levels measured on the day of the procedure were recorded. Sedation was performed using a standardized protocol with midazolam, fentanyl, and propofol titrated to maintain BIS values between 40 and 60. Propofol consumption was normalized to body weight (mg/kg) and procedure duration (μg/kg/min). Correlation analyses and multivariable linear regression models were used to evaluate associations. **Results:** Mean propofol consumption was 157.3 ± 53.1 mg (2.41 ± 0.83 mg/kg), corresponding to an infusion rate of 125.7 ± 69.6 μg/kg/min. In multivariable analysis, estradiol levels were independently associated with propofol requirement (β = 0.238, *p* = 0.014), whereas progesterone levels were not significantly associated with anesthetic dosing after adjustment. BMI (β = −0.305, *p* = 0.002) and procedure duration (β = 0.224, *p* = 0.021) were also identified as independent predictors. **Conclusions:** Estradiol levels were associated with propofol requirement during OPU performed under BIS-guided sedation. However, given the observational design and the modest magnitude of the observed associations, these findings should be interpreted cautiously. BMI and procedure duration appeared to be more consistent predictors of propofol administration.

## 1. Introduction

Oocyte pick-up (OPU) is a key step in in vitro fertilization (IVF) treatment and is typically performed under intravenous sedation or short general anesthesia. Although the procedure is relatively brief, transvaginal follicular aspiration can cause significant visceral discomfort and pain, requiring adequate sedation and analgesia. Propofol-based anesthesia is widely used during OPU because of its rapid onset, short duration of action, and favorable recovery profile [1]. Despite these advantages, substantial interindividual variability in propofol requirement has been observed during OPU procedures, and the factors contributing to this variability remain incompletely understood.

IVF treatment involves controlled ovarian hyperstimulation (COH), which is designed to promote the development of multiple ovarian follicles through administration of exogenous gonadotropins. During COH, granulosa cells produce large amounts of estradiol, resulting in serum estradiol concentrations that are often several-fold higher than those observed in natural menstrual cycles [2]. In addition, progesterone levels may rise as a result of follicular maturation and early luteinization before oocyte retrieval. Consequently, women undergoing IVF frequently present with supraphysiological concentrations of circulating ovarian steroid hormones at the time of OPU.

Sex steroid hormones are known to influence central nervous system function and may modulate both pain perception and anesthetic sensitivity. Estradiol has been shown to enhance neuronal excitability through modulation of glutamatergic transmission and N-methyl-D-aspartate (NMDA) receptor activity, thereby potentially increasing cortical excitability [3]. In contrast, progesterone and its neuroactive metabolite allopregnanolone act as positive modulators of γ-aminobutyric acid type A (GABA-A) receptors, producing sedative and anxiolytic effects similar to those of several anesthetic agents [4]. Through these mechanisms, fluctuations in circulating sex hormone levels may influence anesthetic requirements and responses to sedative drugs.

Clinical observations also support the relationship between hormonal status and anesthetic sensitivity. Previous studies have reported differences in anesthetic responses across phases of the menstrual cycle, suggesting that variations in circulating ovarian hormones may alter anesthetic requirements [5]. In the context of assisted reproduction, serum estradiol levels can reach markedly elevated concentrations during ovarian stimulation, raising the possibility that hormonal status may contribute to variability in anesthetic drug requirements during OPU. However, available evidence on this topic remains limited. A small number of studies have explored the association between estradiol levels and propofol requirements during oocyte retrieval, primarily focusing on the induction dose required to achieve loss of consciousness [6]. Data evaluating the influence of hormone levels on propofol requirements throughout the entire procedure under depth-of-anesthesia monitoring are still scarce.

Therefore, the aim of the present prospective observational study was to evaluate the relationship between preprocedural serum estradiol and progesterone concentrations and propofol requirement during OPU performed under bispectral index (BIS)-guided sedation. In addition, we aimed to investigate whether circulating hormone levels were associated with early hemodynamic changes during anesthesia.

We hypothesized that higher estradiol levels would be associated with increased propofol requirement, whereas progesterone would be associated with reduced anesthetic requirement.

## 2. Materials and Methods

### 2.1. Study Design and Ethical Approval

This prospective observational study was conducted at a single tertiary fertility center between June and December 2025. The study protocol was approved by the institutional ethics committee (Date: 21 May 2025 approval number: 2025/252) and was performed in accordance with the principles of the Declaration of Helsinki. Written informed consent was obtained from all participants before enrollment. The study was registered at ClinicalTrials.gov (Identifier: NCT07378241). The study was designed as a prospective observational investigation and no interventions were introduced to modify routine clinical practice. The investigators did not influence anesthetic management, and all procedures were performed according to the standard anesthesia protocol routinely used in our institution.

### 2.2. Patient Population

Women undergoing oocyte pick-up (OPU) as part of in vitro fertilization (IVF) treatment during the study period were screened for eligibility. Patients were included if they underwent OPU within the predefined study period and had available preprocedural serum estradiol and progesterone measurements obtained on the day of the procedure. Patients were excluded if they had a body mass index (BMI) > 30 kg/m^2^, a history of psychiatric disorders or current use of psychotropic medications, chronic pain syndromes, or missing preoperative hormone measurements.

### 2.3. Anesthesia Protocol

All OPU procedures were performed under intravenous sedation according to the routine anesthesia protocol used in our center. Sedation management was performed by anesthesiologists experienced in assisted reproductive procedures using a standardized protocol. According to the standard institutional protocol, patients routinely receive premedication with midazolam (1 mg) and fentanyl (1 μg/kg). Anesthesia is then induced with propofol at a dose of 1 mg/kg. Following induction, additional bolus doses of propofol are administered and titrated to maintain an adequate depth of sedation.

Standard intraoperative monitoring included electrocardiography, noninvasive blood pressure, pulse oximetry, heart rate, and bispectral index (BIS) monitoring. Sedation depth was additionally assessed clinically using the Ramsay Sedation Scale. Hemodynamic parameters, including blood pressure and heart rate, were monitored throughout the procedure. Sedation was titrated to maintain Ramsay sedation scores between 5 and 6 together with BIS values between 40 and 60. Deep sedation targeting BIS values between 40 and 60 was preferred in order to minimize patient movement and procedural discomfort during transvaginal oocyte retrieval.

Importantly, the investigators did not intervene in anesthetic drug selection, dosing, or titration during the procedures. All anesthetic management decisions were made solely by the attending anesthesiologists according to routine clinical practice. All procedures were performed at a single tertiary fertility center using a standardized institutional sedation protocol.

### 2.4. Data Collection

Demographic and clinical variables including age, body weight, height, body mass index (BMI), and the presence of comorbid diseases were recorded. Preoperative serum estradiol (ng/L) and progesterone (µg/L) levels were measured from blood samples obtained on the morning of the procedure and recorded from the medical records. Procedural variables including total anesthesia duration, procedure duration, and the total doses of midazolam, fentanyl, and propofol administered were also documented.

Hemodynamic parameters including systolic blood pressure, diastolic blood pressure, and heart rate were recorded before induction of anesthesia and at 10, 20, and 30 min after induction. Total propofol consumption was recorded in milligrams and normalized to body weight (mg/kg). In addition, propofol requirement was calculated as micrograms per kilogram per minute (μg/kg/min) by dividing the total administered propofol dose by body weight and procedure duration.

### 2.5. Exposure Variables and Covariates

The primary exposure variables were preprocedural serum estradiol and progesterone concentrations measured on the morning of the OPU procedure. Covariates included age, body mass index (BMI), and procedure duration, which were selected based on their potential clinical association with anesthetic drug requirement and propofol pharmacokinetics.

### 2.6. Outcomes

The primary outcome of the study was intraoperative propofol consumption normalized to body weight (mg/kg). Administered propofol dose was used as a surrogate marker of anesthetic requirement within the context of routine clinical sedation practice. The secondary outcome was the association between hormone levels and early intraoperative hemodynamic changes during anesthesia. In addition, sensitivity analyses were performed using propofol infusion rate normalized to body weight and procedure duration (μg/kg/min).

### 2.7. Statistical Analysis

Continuous variables were assessed for normality using visual methods and the Shapiro–Wilk test. Normally distributed variables are presented as mean ± standard deviation, whereas non-normally distributed variables are presented as median (interquartile range). Spearman correlation analysis was used to evaluate associations between hormonal variables and propofol requirement. Multivariable linear regression analyses were performed to identify independent predictors of intraoperative propofol consumption after adjustment for clinically relevant covariates including age, BMI, and procedure duration. Separate regression models were constructed for estradiol and progesterone because of the potential biological interaction between hormonal variables. Variance inflation factor (VIF) values were used to assess multicollinearity. As exploratory analyses, hormone levels were additionally evaluated for potential non-linear associations. No substantial deviation from linearity was observed. A two-sided *p* value < 0.05 was considered statistically significant. As this was an exploratory prospective observational study and limited prior data were available to estimate the expected effect size, no formal a priori sample size calculation was performed. The sample size was determined by the number of eligible patients undergoing OPU during the predefined study period. Statistical analyses were performed using SPSS version 30.0.0.0 (IBM Corp., Armonk, NY, USA).

## 3. Results

**Figure 1:**
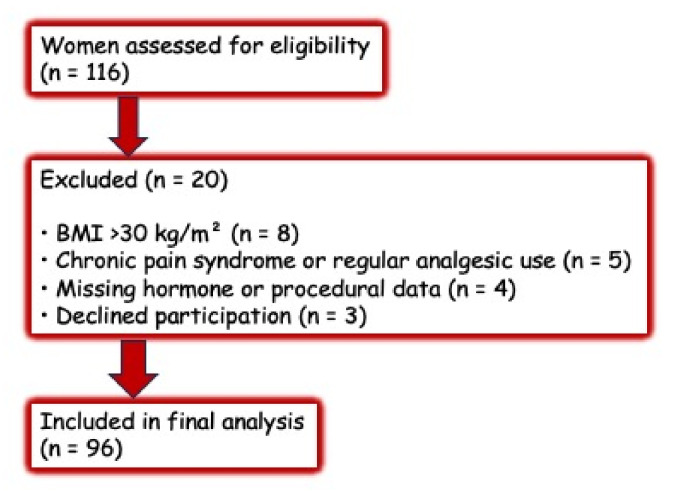
Flow diagram of patient selection and study inclusion.

During the study period, 116 women scheduled for oocyte pick-up were assessed for eligibility. Twenty patients were excluded: eight because of BMI > 30 kg/m^2^, five because of chronic pain syndromes or regular analgesic use, four because of missing hormone or procedural data, and three because they declined participation. Finally, 96 patients were included in the final analysis cohort. The patient selection process is summarized in Figure 1. The mean age was 33.2 ± 4.9 years, and the mean body mass index (BMI) was 24.3 ± 3.1 kg/m^2^. Table 1 summarizes the baseline demographic, hormonal, and procedural characteristics of the overall study cohort. Mean anesthesia duration was 29.9 ± 6.6 min and mean procedure duration was 25.4 ± 6.4 min. The total propofol dose administered was 157.3 ± 53.1 mg (2.41 ± 0.83 mg/kg). When normalized to procedure duration, the mean propofol infusion rate was 125.7 ± 69.6 μg/kg/min.

Spearman correlation analysis demonstrated a weak but statistically significant positive correlation between progesterone levels and propofol requirement (mg/kg) (rho = 0.234, *p* = 0.022), whereas estradiol showed a positive but non-significant correlation (rho = 0.191, *p* = 0.062). The estradiol-to-progesterone ratio was not associated with propofol requirement (rho = −0.037, *p* = 0.720). Propofol requirement was negatively correlated with age (rho = −0.241, *p* = 0.018) and BMI (rho = −0.349, *p* < 0.001). These findings are summarized in Table 2.

Spearman correlation coefficients demonstrating the relationship between hormonal variables, demographic characteristics, and propofol requirement normalized to body weight.

Multivariable linear regression model evaluating independent predictors of Propofol Requirement. Standardized beta coefficients and variance inflation factors (VIF) are presented to assess the strength of association and potential multicollinearity.

In univariable linear regression analyses, BMI, age, and procedure duration were significantly associated with propofol requirement, whereas progesterone and estradiol levels demonstrated borderline associations. In the multivariable model including progesterone, BMI (β = −0.305, *p* = 0.002) and procedure duration (β = 0.224, *p* = 0.021) remained independently associated with propofol requirement, while progesterone was not (*p* = 0.606) (Table 3).

In a multivariable model including estradiol instead of progesterone, estradiol was associated with propofol requirement (β = 0.238, *p* = 0.014), explaining 26.4% of the variance.

Sensitivity analysis using propofol infusion rate (μg/kg/min) as the dependent variable showed that BMI (β = −0.244, *p* = 0.015) remained independently associated with propofol requirement, whereas the strength of the estradiol association varied across models. As an additional sensitivity analysis, a combined multivariable model including both estradiol and progesterone was constructed using propofol infusion rate (μg/kg/min) as the dependent variable. In this model, estradiol remained independently associated with propofol infusion rate (β = 0.170, *p* = 0.026), whereas progesterone was not significantly associated with this outcome (β = −0.061, *p* = 0.414). BMI and procedure duration also remained significant predictors. The results of the sensitivity analysis are presented in Table 4.

No significant associations were observed between hormonal variables and early hemodynamic changes during anesthesia. Detailed associations between estradiol, progesterone, estradiol-to-progesterone ratio, and changes in mean arterial pressure and heart rate are presented in Supplementary Table S1. No significant correlations were also observed between hemodynamic changes and propofol requirement, age, or BMI (all *p* > 0.05).

Multicollinearity analysis showed no relevant concerns, with variance inflation factor (VIF) values ranging from 1.02 to 1.12.

## 4. Discussion

In this prospective observational study, we evaluated the relationship between preprocedural serum estradiol and progesterone levels and propofol requirements during oocyte pick-up performed under BIS-guided sedation. The principal findings of this prospective observational study were that estradiol levels were associated with propofol consumption during oocyte retrieval performed under BIS-guided sedation, whereas progesterone levels were not independently associated with anesthetic dosing. In addition, BMI and procedure duration were identified as more consistent clinical predictors of propofol administration.

Sex steroid hormones have long been recognized as modulators of central nervous system function and may influence anesthetic sensitivity through several neurophysiological mechanisms. Experimental studies have suggested that estrogen may influence neuronal excitability and synaptic transmission [3]. Therefore, elevated estradiol concentrations during controlled ovarian stimulation may potentially contribute to variability in anesthetic responsiveness. However, the observational design of the present study does not allow causal or mechanistic conclusions. In the context of assisted reproductive technologies, this mechanism may become particularly relevant because estradiol concentrations during controlled ovarian hyperstimulation can reach levels several times higher than those observed during natural menstrual cycles [2]. The supraphysiological hormonal environment present during IVF cycles may therefore contribute to variability in anesthetic drug requirements during OPU procedures; however, this hypothesis requires confirmation in dedicated pharmacokinetic and pharmacodynamic studies.

In addition to its effects on neuronal excitability, estrogen has also been shown to modulate nociceptive pathways. Estrogen receptors are widely expressed in several brain regions involved in pain perception, including the hippocampus, amygdala, and brainstem nuclei. Experimental and clinical studies suggest that estrogen may influence both peripheral and central mechanisms of pain modulation, potentially altering pain sensitivity and analgesic requirements [3]. Because oocyte retrieval involves transvaginal follicular aspiration, which can produce visceral discomfort and nociceptive stimulation, hormonal modulation of pain pathways may represent an additional mechanism contributing to variability in propofol requirement in this setting.

Our findings are partly consistent with previous clinical observations regarding hormonal influences on anesthetic requirements. Basaran et al. reported a positive correlation between serum estradiol levels and propofol consumption during oocyte retrieval procedures [7]. However, their study primarily focused on the propofol dose required to achieve loss of consciousness. In contrast, our study evaluated anesthetic requirements throughout the entire procedure using BIS-guided titration, which may provide a more comprehensive assessment of anesthetic demand during OPU procedures. Continuous monitoring of sedation depth allows more accurate adjustment of anesthetic dosing and may therefore provide a more comprehensive assessment of propofol administration during OPU procedures.

In contrast to estradiol, progesterone levels were not independently associated with propofol requirement in our multivariable analyses. Progesterone and its neuroactive metabolites are known positive modulators of γ-aminobutyric acid type A (GABA-A) receptors and are therefore thought to potentially exert sedative and anxiolytic effects through enhancement of inhibitory neurotransmission [4]. Consistent with this mechanism, several studies have suggested that anesthetic requirements may vary across the menstrual cycle. For example, Fu et al. reported that the propofol EC50 for inducing loss of consciousness was lower during the luteal phase, when progesterone levels are typically higher [5]. Similarly, Zhou et al. demonstrated that dexmedetomidine sedation produced deeper BIS reductions during the luteal phase compared with the follicular phase [6]. However, the independent clinical influence of circulating progesterone levels on anesthetic drug requirements remains inconsistent. In the setting of controlled ovarian stimulation, estradiol and progesterone levels may increase simultaneously, and the excitatory effects of elevated estradiol may partially offset the sedative properties of progesterone, potentially explaining the absence of an independent progesterone effect in our analysis.

In addition to hormonal variables, BMI was identified as an independent predictor of propofol requirement in our study, with higher BMI values associated with lower weight-adjusted propofol dosing. This observation may be explained by the pharmacokinetic characteristics of propofol, a highly lipophilic anesthetic agent that distributes extensively into adipose tissue. Previous pharmacokinetic studies have suggested that increasing body mass index may alter the pharmacokinetic and pharmacodynamic properties of propofol, potentially influencing weight-adjusted anesthetic requirements. [8]. Furthermore, population pharmacokinetic modelling studies including patients across a wide BMI spectrum have shown that body weight descriptors such as total body weight and lean body weight significantly influence propofol clearance and distribution volumes, highlighting the important role of body composition in determining propofol pharmacokinetics [9]. Together, these findings support the notion that alterations in body composition may contribute to variability in weight-adjusted propofol requirements among patients with different BMI values.

Another notable finding of our study is the absence of a significant association between hormonal variables and early hemodynamic responses during anesthesia. Neither estradiol nor progesterone levels were correlated with changes in mean arterial pressure or heart rate during the procedure. These findings suggest that although hormonal status may influence anesthetic drug requirements, it does not appear to significantly affect early hemodynamic stability during propofol sedation in patients undergoing OPU.

The observed hormonal associations were modest in magnitude, whereas BMI and procedural factors demonstrated more consistent associations with propofol administration. Therefore, the present findings should be interpreted cautiously and primarily considered hypothesis-generating.

The present study has several strengths. First, the prospective observational design allowed systematic data collection without interfering with routine clinical practice. Second, sedation depth was standardized using BIS monitoring, enabling objective titration of propofol during the entire procedure. Third, our study evaluated anesthetic requirements throughout the entire OPU procedure rather than focusing solely on induction doses, which may provide a more comprehensive understanding of anesthetic demand in this clinical setting.

Several limitations should also be acknowledged. The study was conducted at a single center and included a relatively limited sample size. Hormone levels were measured at a single time point on the day of the procedure and may not fully reflect dynamic hormonal fluctuations during ovarian stimulation. Furthermore, potential confounding factors including procedural nociceptive stimulation, patient anxiety and inter-anesthesiologist differences may also have influenced administered propofol dose and were not fully controlled in the present observational design.

We acknowledge that administered propofol dose represents a surrogate marker of anesthetic requirement and may partly reflect anesthesiologist titration behavior and clinical sedation strategies rather than intrinsic pharmacodynamic anesthetic sensitivity. More detailed pharmacokinetic and pharmacodynamic studies incorporating plasma propofol concentrations or effect-site modeling would be required to clarify whether reproductive hormones directly influence anesthetic sensitivity.

Although BIS values between 40 and 60 are commonly associated with general anesthesia, similar targets may also be used during deep sedation for oocyte retrieval procedures to optimize patient immobility and procedural conditions. Nevertheless, the selected sedation depth target may have influenced total propofol administration and should be considered when interpreting the findings.

Accordingly, the present findings should not be interpreted as direct evidence of altered pharmacodynamic anesthetic sensitivity.

From a clinical perspective, our findings suggest that hormonal status, particularly estradiol levels, may contribute to interindividual variability in propofol requirements during OPU procedures. Given the markedly elevated estradiol concentrations observed during controlled ovarian stimulation, anesthesiologists should be aware that hormonal factors may influence anesthetic drug requirements in patients undergoing IVF treatments. Future studies with larger patient populations and pharmacokinetic–pharmacodynamic modeling approaches may help further clarify the relationship between reproductive hormones and anesthetic drug requirements.

In conclusion, estradiol levels were associated with propofol consumption during oocyte retrieval performed under BIS-guided sedation. However, given the observational design and the use of administered propofol dose as a surrogate marker of anesthetic requirement, these findings should be interpreted cautiously. Clinical and procedural variables, particularly BMI and procedure duration, appeared to have more consistent associations with propofol administration. Further pharmacokinetic and pharmacodynamic studies are needed to clarify whether reproductive hormones directly influence anesthetic sensitivity.

## Figures and Tables

**Table 1:** Baseline demographic, hormonal, and procedural characteristics of the study population.

Variable	Value
Age (years)	33.2 ± 4.9
BMI (kg/m^2^)	24.3 ± 3.1
Progesterone (µg/L)	4.23 (IQR 5.77)
Estradiol (ng/L)	776 (IQR 1096)
Estradiol/Progesterone ratio	129.4 (IQR 162.5)
Number of retrieved oocytes	7.8 ± 7.0
Anesthesia duration (min)	29.9 ± 6.6
Procedure duration (min)	25.4 ± 6.4
Total propofol dose (mg)	157.3 ± 53.1
Propofol dose (mg/kg)	2.41 ± 0.83
Propofol infusion rate (mcg/kg/min)	125.7 ± 69.6

Baseline demographic characteristics, hormone levels, and procedural variables of the study population undergoing oocyte pick-up under propofol sedation. Continuous variables are presented as mean ± standard deviation or median (interquartile range) where appropriate.

**Table 2:** Correlation Analysis with Propofol Requirement (mg/kg).

Variable	Spearman rho	*p* Value
Progesterone (µg/L)	0.234	0.022
Estradiol (ng/L)	0.191	0.062
Estradiol/Progesterone ratio	−0.037	0.720
Age	−0.241	0.018
BMI	−0.349	<0.001

**Table 3:** Multivariable Linear Regression for Propofol Requirement (mg/kg).

Predictor	Standardized Beta	*p* Value	VIF
BMI	−0.305	0.002	1.05
Procedure duration	0.224	0.021	1.08
Age	−0.183	0.059	1.02
Progesterone	0.051	0.606	1.10

**Table 4:** Sensitivity Analysis: Multivariable Linear Regression for Propofol Infusion Rate (mcg/kg/min).

Predictor	Standardized Beta	*p* Value	VIF
BMI	−0.244	0.015	1.06
Estradiol	0.254	0.014	1.09
Age	−0.101	0.320	1.03

Sensitivity analysis evaluating predictors of propofol infusion rate when normalized to procedure duration. Standardized beta coefficients and VIF values are reported. The combined estradiol–progesterone model is described in the text.

## Data Availability

The data presented in this study are available from the corresponding author upon reasonable request.

## Supplementary Materials

The following supporting information can be downloaded at https://www.mdpi.com/article/10.3390/jcm15114280/s1. Table S1: Associations between hormonal variables and early intraoperative hemodynamic changes during oocyte retrieval procedures.
