# Supplementary material for: Clinical and Hormonal Determinants of Propofol Requirement During Oocyte Pick-Up: A Prospective Observational Study
Source: J Clin Med. 2026 Jun 1;15(11):4280. doi: 10.3390/jcm15114280 (PMC13258623; doi:10.3390/jcm15114280)
Supplement: Supplementary file 1 [file jcm-15-04280-s001.zip › jcm-4335483-supplementary.pdf]

Supplementary Materials:

Supplementary Table S1. Associations between hormonal variables and early intraoperative hemodynamic changes during oocyte retrieval procedures.

**Hormonal Variable**	**Outcome**	**B coefficient (95% CI)**	**p value**
Estradiol	DeltaMAP10	−0.001 (−0.003 to 0.002)	0.605
Estradiol	DeltaMAP15	−0.001 (−0.003 to 0.002)	0.533
Estradiol	DeltaHR10	0.000 (−0.001 to 0.002)	0.829
Estradiol	DeltaHR15	0.000 (−0.002 to 0.002)	0.861
Progesterone	DeltaMAP10	0.183 (−0.262 to 0.628)	0.420
Progesterone	DeltaMAP15	−0.058 (−0.552 to 0.436)	0.818
Progesterone	DeltaHR10	0.097 (−0.186 to 0.380)	0.501
Progesterone	DeltaHR15	−0.031 (−0.434 to 0.372)	0.881
EP ratio	DeltaMAP10	−0.001 (−0.003 to 0.002)	0.546
EP ratio	DeltaMAP15	−0.001 (−0.003 to 0.002)	0.591
EP ratio	DeltaHR10	0.000 (−0.001 to 0.002)	0.795
EP ratio	DeltaHR15	0.001 (−0.001 to 0.003)	0.383

Abbreviations: CI, confidence interval; MAP, mean arterial pressure; HR, heart rate; EP ratio, estradiol-to-progesterone ratio.

DeltaMAP10 and DeltaMAP15 represent changes in mean arterial pressure at 10 and 15 minutes after induction of sedation, respectively. DeltaHR10 and DeltaHR15 represent changes in heart rate at 10 and 15 minutes after induction of sedation, respectively.

B coefficients were obtained from univariable linear regression analyses.
